# Shifting perspectives in coronary involvement of polyarteritis nodosa: case of 3-vessel occlusion treated with 4-vessel CABG and review of literature

**DOI:** 10.1186/s12872-024-03841-y

**Published:** 2024-04-02

**Authors:** Dylan J. Walter, Grace E. Bigham, Steven Lahti, Syed W. Haider

**Affiliations:** 1 Department of Internal Medicine, MedStar Georgetown University Hospital, Washington DC, 20007 USA; 2 Cardiovascular Diseases, MedStar Washington Hospital Center, Washington DC, 20010 USA; 3 Division of Cardiovascular Sciences, , USF Morsani College of Medicine, 2 Tampa General, Circle, STC 5Th Floor, Tampa, Fl 33606 USA

**Keywords:** Polyarteritis nodosa, Coronary artery disease, PCI in coronary arteritis, CABG in coronary arteritis, Systemic vasculitis and coronary artery disease

## Abstract

**Background:**

Polyarteritis Nodosa (PAN) is a systemic vasculitis (SV) historically thought to spare the coronary arteries. Coronary angiography and contemporary imaging reveal coronary stenosis and dilation, which are associated with significant morbidity and mortality. Coronary arteries in PAN are burdened with accelerated atherosclerosis from generalized inflammation adding to an inherent arteritic process. Traditional atherosclerotic risk factors fail to approximate risk. Few reports document coronary pathology and optimal therapy has been guarded.

**Methods:**

Database publication query of English literature from 1990–2022.

**Results:**

Severity of coronary involvement eludes laboratory monitoring, but coronary disease associates with several clinical symptoms. Framingham risk factors inadequately approximate disease burden. Separating atherosclerosis from arteritis requires advanced angiographic methods. Therapy includes anticoagulation, immunosuppression and revascularization. PCI has been the mainstay, though stenting is confounded by vagarious alteration in luminal diameter and reports of neointimization soon after placement.

**Conclusions:**

When graft selection avoids the vascular territory of SV’s, CABG offers definitive therapy. We have contributed report of a novel CABG configuration in addition to reviewing, updating and discussing the literature. Accumulating evidence suggests discrete clinical symptoms warrant suspicion for coronary involvement.

## Highlights

- Polyarteritis Nodosa causes coronary arteritis, commonly in asymptomatic patients

- Angiography with Optical Coherence Tomography or ultrasound differentiates the disease

- Laboratory monitoring does not correlate with coronary severity

- Coronary involvement is associated with abdominal pain, new hypertension and young age

- Standard atherosclerotic risk factors do not associate with coronary involvement

- PCI is complicated by arterial stenosis adjacent to dilation and rapid neo-intimal expansion

- CABG offers definitive therapy, though selection of graft material is complex

## Introduction

Coronary artery disease (CAD) is potentiated by systemic inflammation, evidenced by earlier onset and increased incidence among chronic inflammatory and autoimmune conditions, such as rheumatoid arthritis, systemic lupus, and HIV [1]. CAD in primary systemic vascular diseases (PSV) is well-documented and inherent to both a generalized inflammatory state and the underlying disease mechanism itself [2, 3]. Systemic disease may be present in up to 20% of young patients (< 40y) with premature and advanced CAD [4].

Polyarteritis nodosa (PAN) is a medium vessel necrotizing vasculitis causing intimal proliferation, luminal narrowing and potential for thrombosis, ischemia or infarction. Vessel wall inflammation may contribute to vessel aneurysm, dissection and even rupture [2, 5, 6]. Severe disease entails renal involvement, mononeuritis multiplex, muscle or mesenteric involvement, limb or digit ischemia and coronary involvement [7]. Individuals with necrotizing arteritis have significantly higher 5-year mortality compared to those without cardiac manifestation [8]. Yet, few reports have characterized cardiac manifestations of PAN, with most cases being discovered incidentally or during post-mortem analysis [5, 9, 10].

An annual incidence of 0–1.6 cases per million persons, onset predominantly in the 5th or 6th decade and prevalence of ~ 31 cases per million persons in European countries besets characterization of PAN as an orphan disease [4]. Effects on coronary vasculature are under-represented, though stenosis, ectasia, aneurysm, dissection, rupture, and sudden cardiac death are documented [2, 5, 6]. Historically, coronary disease in PAN was thought rare and less significant compared to the burdens of cutaneous, renal, neurologic, and mesenteric pathology. Recent literature suggests coronary involvement is more common than previously thought. Moreover, retrospective data points to younger age (age < 40), celiac involvement and new-onset hypertension as strong predictors [4]. Treatment modalities are convoluted owing to reports of neo-intimal growth in implanted stents causing re-stenosis and early post-operative rupture of vasculature in surgical patients [11, 4]. Consequently, sparsity in literature documenting successful coronary bypass in this population exists.

Herein, we summarize recent updated literature offering perspective on therapeutic strategy for coronary PAN. Additionally, we present a patient with chronic epigastric pain and new diagnosis of PAN found to have 3-vessel occlusive disease treated successfully with 4-vessel CABG.

## Methods

PubMEd query filtered for English literature from 1990 – 2022. Query phrases “Polyarteritis Nodosa and Coronary Artery Disease” and “Polyarteritis Nodosa and Coronary Artery Bypass Grafting” resulted in 29 and 8 hits, respectively. Literature hits with primary focus devoted to polyarteritis and coronary artery disease in adults were reviewed. References for each study selected were screened. Additional articles known to the authors relevant to coronary arteritis not contained in key search phrases were included in discussion.

### Our case

**Fig. 1:**
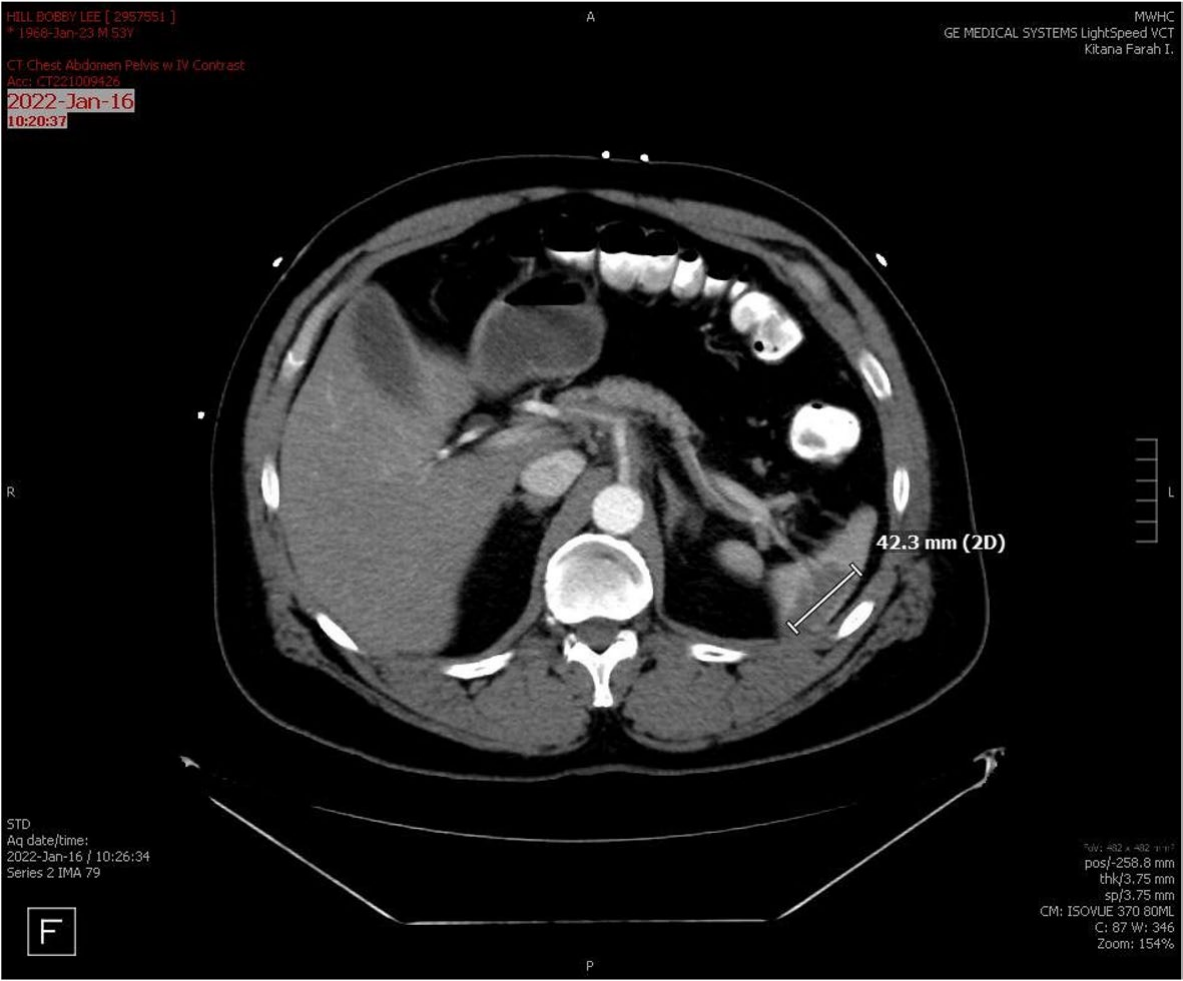
CT Abdomen – splenic infarction, hypoechoic lesion measured at 4.2 cm in length

A 53-year-old African American male with history of hypertension, hyperlipidemia, former tobacco use (20-pack year history), asthma, and obesity presented to the internal medicine clinic with sharp chest pain for 6 months. The pain “came and went" without relationship to exertion but worsened at night and was triggered by eating. Associated shortness of breath, with no response to albuterol inhaler was noted. He was prescribed proton pump inhibitor with isosorbide mononitrate and scheduled for an outpatient stress test. That week, he developed unrelenting epigastric and left upper quadrant abdominal pain and presented to the emergency department where abdominal CT without contrast revealed splenic infarction (Fig. 1). EKG showed sinus rhythm without segment or ST abnormalities. Laboratory workup was remarkable for elevated inflammatory markers: ESR 33 and hsCRP 14 mg/L. His troponin level, urinalysis and infectious work-up, including hepatitis panel and hepatitis B serology were unremarkable.

**Fig. 2:**
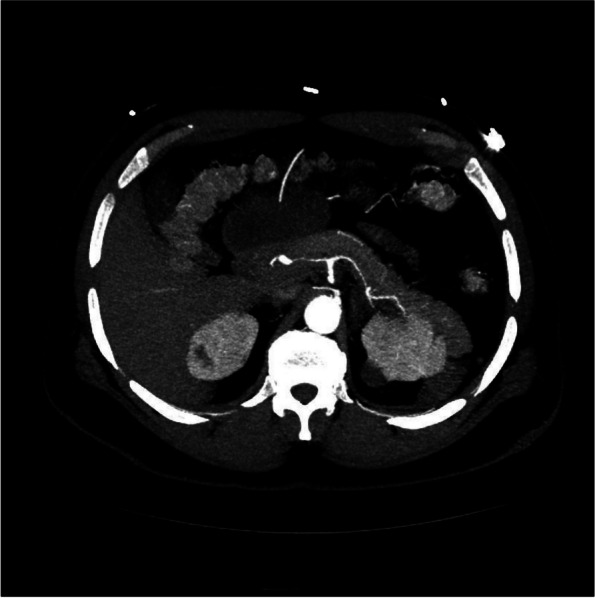
CT Angiography – Narrowing and Dilation of the celiac axis and proximal branches

**Fig. 3:**
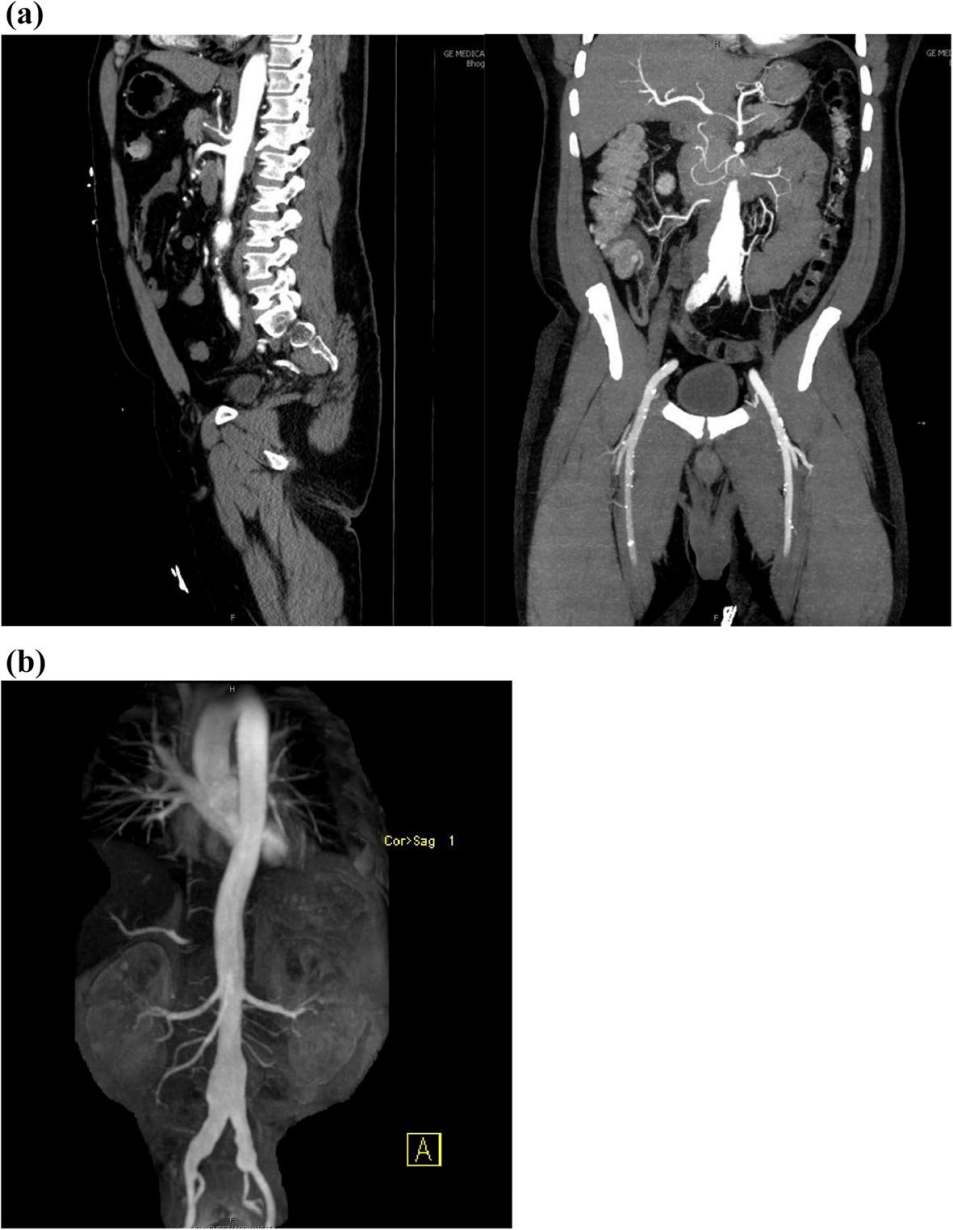
**a** CT Angiography – Abdominal Aortic Aneurysm, Celiac Trunk and Proximal branch narrowing (**b**) MRA – Abdominal Aortic Aneurysm and bilateral Femoral Artery Aneurysm

He was admitted for evaluation and had a negative hypercoagulability and polycythemia workup including D-dimer, Ferritin, APLS, Factor V Leiden assay, Prothrombin gene mutation, antithrombin III activity, homocysteine, protein C and S, ANA, JAK2, EPO, Hepatitis and HIV testing. However, episodic bouts of retrosternal chest pain unresponsive to nitroglycerin continued. Serial EKGs were negative for ischemic changes. The surgical team pursued contrast CT for operative planning which uncovered luminal narrowing of the celiac axis and its major branches (proximal hepatic and splenic arteries) (Figs. 2 and 3a). CT also revealed a 3.3 cm saccular aneurysm of the abdominal aorta above the bifurcation with additional aneurysmal abnormalities of the right (measuring 1.8 cm) and left Iliac artery (measuring 2 cm diameter) (Fig. 3). Rheumatology evaluated for vasculitis; but felt the distribution of vascular lesions were inconsistent with typical PAN referencing limited celiac involvement with only initial segments of the splenic and hepatic arteries being affected. He underwent trans-thoracic echocardiography (TTE) which demonstrated a hyperdynamic left ventricle with an estimated ejection fraction of 65–70% and mild hypokinesis of inferior and infero-septal basal segments. Further testing for ANA, MPO, PR-3 and RF was negative but C3 and C4 levels were elevated. A joint radiology-rheumatology conference to decide further management steps, sent the patient for MRI/MRA which revealed persistent narrowing and inflammation of the celiac, splenic and common hepatic artery with pre-contrast T1 hyperintensity and post-contrast enhancement in the arterial wall. IV methylprednisolone was subsequentially initiated.

Episodic bouts of chest pain persisted without EKG change; repeated troponins remained negative. Formal cardiac evaluation for inferior wall motion abnormalities (WMA) was pursued. Cardiac MRI showed LV concentric hypertrophy with normal function, no wall motion abnormalities and absence of late gadolinium enhancement or other signs of infiltrative disease, ischemia or fibrosis. He completed a 4-day course of methylprednisolone (60 mg IV) and was discharged on oral prednisone (60 mg PO) with an unconfirmed diagnosis of PAN.

In the outpatient setting, he underwent PET scan, notably unremarkable. Repeat CTA confirmed luminal narrowing of the common hepatic and proximal splenic arteries, however, now with new radiologic evidence of a beading appearance in the SMA and celiac trunk. With the combination of laboratory and imaging findings, PAN was determined to be the unifying clinical diagnosis. Mesenteric involvement raised concerns for advanced disease and motivated the decision to proceed with cyclophosphamide (Cyc) infusion therapy.

**Fig. 4:**
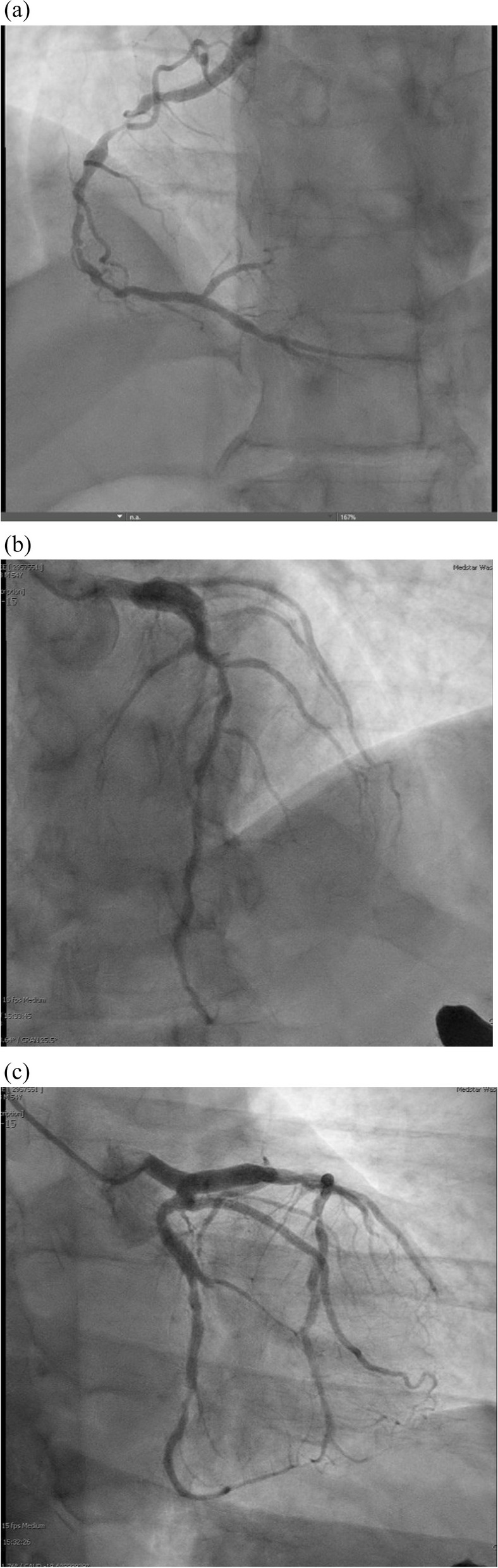
Coronary Angiography – (**a**) RCA, (**b**) LAD and (**c**) LCx – Each coronary artery showed evidence of diffuse, alternating stenosis and dilatation

Prior to Cyc initiation, unrelenting epigastric and left upper quadrant abdominal pain prompted presentation. Again, EKG and troponin levels were negative. Most features of the pain episodes aligned with prior, including intermittent nature, association with food consumption, and absent response to nitroglycerin. However, he now had new onset bilateral numbness in his hands and feet. Inflammatory markers showed CRP 5 (4 at discharge) and ESR 49 (8 at discharge). Esophageal x-ray was negative for abnormality; upper endoscopy for mucosal ulceration was unrevealing. Coronary CT was pursued and showed severe coronary calcification with Agatston score of 981 (99th percentile), preventing analysis of stenosis. Left heart catheterization (LHC) revealed 3-vessel disease, with 90% stenosis of the LAD, 70% of the 2nd diagonal, 100% of the obtuse marginal 1, 70% of the circumflex and 90% RCA occlusion (Fig. 4). He was discharged on antianginals with referral for CABG.

Five days post-discharge, successful 4-vessel CABG with skeletonized LIMA technique ( LIMA-LAD and SVG-OM-LPDA-RPDA) was completed under 89 min of bypass time; the 2nd diagonal was small rendering inability to bypass. Preoperatively, LIMA flow was excellent; Intraoperatively, 100 mg hydrocortisone was given for his subacute history of prednisone therapy. His Postoperative surgical graft flow was excellent with easy separation from bypass and and echocardiogram following the procedure showed LVEF 60%. Discharge was post-operative day 7, with medications including aspirin, clopidogrel, metoprolol, rosuvastatin, olmesartan and referral for cardiac rehabilitation. Medical management of PAN included daily prednisone and follow-up with rheumatology to determine optimal timing for initiation of cyclophosphamide while recovering.

## Discussion

Since the inauguration of literature covering PAN coronary arteritis, report of CABG has been rare and successful surgical revascularization in patients with diffuse 3-vessel arteritis is limited to 3 cases (Table 4) [7]. PCI presently encompasses the mainstay of treatment but low case numbers, difficulty in stent placement related to erratic luminal diameter and report of neo-intimal media expansion into apposed stents leave unsettled the optimal revascularization strategy [4, 7, 11, 12]. A minimally invasive strategy is reasonable in patients with 1 or 2-vessel disease but owing to the mechanism of PAN as a PSV, surgical revascularization, when composed of graft material having less propensity for the systemic pathophysiology of PAN than the coronary arteries, offers a definitive strategy to patients with diffuse 3-vessel disease. In the first case of a 4-vessel CABG configured LIMA-LAD and SVG-OM-LPDA-RPDA, we report excellent post-operative patency and flow.

More broadly, PSVs are categorized by the distribution of vasculature affected (i.e., small, medium, and large). Historically, PSVs are thought to spare the coronary arteries, though recent contributions report 10–50%, 10–45% and 25–30% rates of coronary involvement with PAN, Takayasu and Kawasaki’s vasculitis, respectively [13]. PAN, first described in 1866, is a PSV causing inflammation in medium-sized vessels, with infrequent small vessel involvement [14]. Literature first discusses known PAN coronary arteritis in 1948 [15]. Associations of congestive heart failure, hypertension, pericarditis, and arrhythmia are well documented; yet the bulk of literature and teaching suggest severe coronary disease is rare. Recent case reports and the first retrospective cohort study now argue this point. Furthermore, with modern interventional therapies, a summation of outcomes is long due.

Following histopathologic and clinical associations between PAN and coronary disease, several sought to characterize coronary pathology. Post-mortem analysis of 66 PAN cases by Holsinger and colleagues revealed arteritis in 41 patients (62%); among which presenting symptoms are displayed in Table 1 [9]. Overall, 89% of hearts with MI pathology also had arteritis [9]. A separate team conducted necropsy of 36 patients with PAN reporting 18 (50%) had histological evidence of coronary involvement [10]. (Cassling et al.) tabulated cumulative autopsy confirmed coronary diseased PAN cases in 1985, leading those after him to posit typical coronary syndrome symptoms were rare opposite of systemic hypertension, heart failure, and renal failure, the most common presenting symptoms (Table 1) [16]. Cumulatively, the mean survival in untreated individuals with coronary involvement was 8 months contrasting 5 years in treated individuals [16].

**Table 1:** Frequency of coronary events and symptoms at presentation in prior literature

	**Myocardial Infarction**	**Coronary Stenosis**	**Coronary Aneurysm**	**Coronary Ectasia**	**Coronary Dissection**	**Common signs and symptoms at presentation**
**Holsinger** (post-mortem study, 1962) [9]	41/66 (62%)	41/66 (62%), coronary arteritis	2/66 (3%)	–	–	Hypertensio, tachycardia, dyspnea
**Schrader** (post-mortem study, 1985) [10]	3/36 (17%)	18/36 (50%), coronary arteritis	–	–	–	Systemic hypertension
**Huang (**February 2021) [12]	15/17 (88%) (EKG, elevated myocardial enzymes)	27/34 (79%)	12/34 (35%) (8/34 multiple)	–	2/34 (5.9%)	Chest pain, dyspnea
**Lai** (June 2021) [4]	8/19 (42%)	14/19 (74%)	4/19 (21%)	1/19 (5.3%)	–	Fever, myalgia, new-onset hypertension
**Total**	67/138 (49%)	100/155 (65%)	18/119 (15%)	1/19 (5.3%)	2/34 (5.9%)	

The pathologic mechanism of coronary arteritis is characterized by three phases (acute, healing, healed), based on histological change. The acute phase is marked by fibrinoid necrosis of the vessel wall and an associated mixed inflammatory infiltrate causing disruption of the media and internal elastic lamina. As vessel wall necrosis and inflammation progresses, perivascular structures are affected. partitioning of inflammatory infiltrates allows fibroblast intrusion, marking the onset of healing. These cells’ products culminate in healed lesions, evidenced by fibrosis, calcium deposition and narrowing of the vessel lumen [16, 17]. Medial invasion and disruption are culprits for vascular aneurysm and thrombosis (acute) while fibroblast proliferation generates luminal stenosis. High degrees of vasculitic stenosis present risk of diagnostic mistake for atherosclerosis, differentiation is a challenge [11].

The acute pathology of coronary PAN (myocardial infarction, dissection, rupture) has been demonstrated in case reports of living patients. Most reports align with our understanding of disease progression as described above. Though limited reports have called into question the mechanisms underlying symptom development, such as (Rajini et al.’s) case of a patient suffering massive anterior wall myocardial infarction (MI) despite angiographically clean vessels and myocardial biopsy without signs of myocarditis. Ultimately, concluding with the postulation that coronary vasospasm underlies some cardiac manifestations of PAN [18]. (Harada et al.) similarly offers a case of MI with non-obstructed coronary arteries (MINOCA) by angiography causing sudden cardiac death, after which autopsy confirmed absence of coronary disease [19]. Across the general population, MINOCA rates reach 25% in persons under 35 and decrease with age. Since true incidence of coronary PAN is unclear, these reports may indicate coronary PAN can cause MI even when stenosis, aneurysm, dissection, and thrombus are absent.

More recently, (Huang et al.) reviewed 34 cases mentioning cardiac involvement in PAN and reported the symptoms at presentation (Table 1) [12]. Additional points contesting the 1985 report, along with coronary event rates are summarized in (Table 1) [7, 12, 16]. Lesion characterization by angiography was available for 23 patients while 14 had autopsy. Table 1 shows the incidence of stenosis, aneurysm & dissection. Seventy-nine percent (79%, 27) of the patients had coronary stenosis, with single vessel disease being most common (11/27, 40%) (RCA 15%, LAD 12%) and diffuse 3-vessel disease least common (7/27, 25.9%). Significance of vessel distribution remains vague when comparing a recent retrospective study that identified 19 patients with coronary lesions [4]. Among these, 1 and 3-vessel disease were most frequent (8/19, 42% & 8/19, 42% respectively) followed by 2-vessel disease (3/19, 16%) [4]. 15 (79%), 14 (74%) and 9 (47%) cases involved the LAD, RCA and LCX, respectively.. The severity of coronary PAN was reflected by 50% (15/30) mortality in a mean 8-month follow-up; speaking to unmet needs for additional work discernably impacting patient outcomes [12].

Our patient had atypical anginal, epigastric and dyspneic symptoms, aligning with frequent symptomatology cited by prior reports (Table 1). His CAG showed 3-vessel disease of the LAD, Circumflex and RCA, again consistent with prior work and harmonious with the fact that PAN is a systemic disease affecting vascular territories globally.

Complicating screening and disease recognition, coronary PAN appears to establish and progress in patients who are either undiagnosed or lacking “classic” systemic manifestations of PSV’s. (Huang et al.) calculated only 26.5% of patients were diagnosed or known to have PAN when presenting with cardiac symptoms [12]. Others highlighted a patient developing new coronary lesions within a 5-day period [5]. Our patient was asymptomatic for several years, developed insidious anginal and abdominal pain for 6 months, and subsequently suffered acute symptom exacerbation as acute splenic infarction, culminating in his diagnosis. Less than 2 months passed between diagnosis and onset of dyspnea, his cardiac investigations and intervention. Further entangling recognition, patients with otherwise “stable,” non-cardiac PAN have developed coronary arteritis in the absence of symptoms or elevated inflammatory markers (CRP, ESR) preventing intervention and risk reduction therapies [12, 20–22]. Retrospective study revealed that only ~ 52% of patients with coronary PAN had elevated ESR and/or CRP [4]. Likewise, other works showed clinical and laboratory methods are often unsuccessful in predicting new findings/positive imaging across visceral organ systems, a theme seemingly reflected in coronary PAN [23]. Incongruence relating to cardiac disease burden and clinical indicators underscores calls for increased surveillance among at risk patients, though the question of whom to screen is one we are only beginning to unravel [4, 12, 24, 25].

The American College of Rheumatology (ACR) guidelines define the diagnosis of PAN by presence of (at least) 3 diagnostic criteria (Table 2) [26]. No routine surveillance or imaging is recommended during clinically quiescent disease (Table 3) [7]. In lieu of standardized screening, the first retrospective study of risk factors was conducted [4]. In the cohort of 145 PAN patients, 19 had coronary involvement. Remarkable findings between patients with and without coronary disease included the absence of significant difference in standard atherosclerosis risk factors (smoking, hypertension, diabetes, hyperlipidemia),. while multi-variate analysis revealed new-onset hypertension (OR 6.668, 95% CI, *P* = 0.003) and celiac artery involvement (OR 3.722, 95% CI, *P* = 0.003) carry significant risk for coronary involvement. Both features (Figs. 2 and 3a) were prominent in our case. Contrarily, weight loss was a protective factor for coronary disease while coronary disease itself increased risk of cranial, carotid, renal, celiac and lower extremity disease [4]. Despite sample size, (Lai et al.’s) study is the first to ascribe significance to risk factors. Knowledge of risk factors and discrepancies between symptoms and/or lab markers with development and progression of coronary arteritis should encourage physicians to consider screening with imaging in defined populations.

**Table 2:** 1990 criteria for the classification of polyarteritis nodosa (American College of Rheumatology) [26]

**Criterion**^a^		**Definition**
1	Weight loss ≥ 4 kg	Loss of 4 kg or more of body weight since illness began, not due to dieting or other factors
2	Livedo reticularis	Mottler reticular pattern over the skin of portions of the extremities or torso
3	Testicular pain or tenderness	Pain or tenderness of the testicles, not due to infection, trauma, or other causes
4	Myalgias, weakness or leg tenderness	Diffuse myalgias (excluding shoulder and hip girdle) or weakness of muscles or tenderness of leg muscles
5	Mononeuropathy or polyneuropathy	Development of mononeuropathy, multiple mononeuropathies, or polyneuropathy
6	Diastolic BP > 90 mmHg	Development of hypertension with the diastolic BP higher than 90 mmHg
7	Elevated BUN or creatinine	Elevation of BUN > 40 mg/dL or creatinine > 1.5 mg/dL, not due to dehydration or obstruction
8	Hepatitis B Virus	Presence of hepatitis B surface antigen or antibody in serum
9	Arteriographic abnormality	Arteriogram showing aneurysms or occlusions of the visceral arteries, not due to arteriosclerosis, fibromuscular dysplasia, or other non-inflammatory causes
10	Biopsy of small or medium-sized artery containing PMN	Histologic changes showing the presence of granulocytes or granulocytes and mononuclear leukocytes in the artery wall

*BP* Blood pressure, *BUN* Blood urea nitrogen, *PMN* Polymorphonuclear neutrophils

^a^For classification purposes, a patient shall be said to have polyarteritis nodosa if at least 3 of these 10 criteria are present. The presence of any 3 or more criteria yields a sensitivity of 82.2% and a specificity of 86.6%

**Table 3:** 2021 American college of rheumatology/vasculitis foundation guideline for the management of polyarteritis nodosa: summary of recommendations [7, 27]

Recommendations/statements for the management of Polyarteritis Nodosa (PAN)	
**Vascular imaging, tissue biopsy and diagnostic testing**	For patients with suspected PAN, we conditionally recommend using abdominal vascular imaging to aid in establishing a diagnosis and determining the extent of disease
	For patients with a history of severe PAN with abdominal involvement who become clinically asymptomatic, we conditionally recommend follow-up abdominal vascular imaging. Indefinite routine vascular imaging should be avoided if the abdominal vascular disease is shown to be quiescent
	For patients with suspected PAN involving the skin, we conditionally recommend obtaining a deep-skin biopsy specimen (i.e., a biopsy reaching the medium-sized vessels of the dermis) over a superficial skin punch biopsy to aid in establishing a diagnosis
	For patients with suspected PAN and peripheral neuropathy (motor and/or sensory), we conditionally recommend obtaining a combined nerve and muscle biopsy over a nerve biopsy alone to aid in establishing a diagnosis
	For patients with a history of peripheral motor neuropathy secondary to PAN, we conditionally recommend serial neurologic examinations instead of repeated electromyography/ nerve conduction studies (e.g., every 6 months) to monitor disease activity
**Treatment of active disease**	For patients with newly diagnosed active, severe PAN, we conditionally recommend initiating treatment with cyclophosphamide and either pulse IV GCs or high-dose oral GCs over high-dose GCs alone
	For patients with newly diagnosed active, severe PAN who are unable to tolerate cyclophosphamide, we conditionally recommend treating with other non-GC immunosuppressive agents and GCs over GCs alone
	In patients with newly diagnosed active, severe PAN, we conditionally recommend against using plasmapheresis combined with cyclophosphamide and GCs over cyclophosphamide and GCs alone
	For patients with newly diagnosed active, non-severe PAN, we conditionally recommend treating with non-GC immunosuppressive agents (MTX or AZA) and GCs over GCs alone
	For patients with PAN in remission who are receiving non-GC immunosuppressive therapy, we conditionally recommend discontinuation of non-GC immunosuppressive agents after 18 months over continued (indefinite) treatment
**Treatment of refractory disease**	For patients with severe PAN that is refractory to treatment with GCs and non-GC immunosuppressive agents other than cyclophosphamide, we conditionally recommend switching the non-GC immunosuppressive agent to cyclophosphamide, over increasing GCs alone
**Remission maintenance**	For patients with newly diagnosed PAN who have achieved disease remission with cyclophosphamide, we conditionally recommend transitioning to another non-GC immunosuppressive agent over continuing cyclophosphamide
**Other considerations**	For patients with PAN with nerve and/or muscle involvement, we conditionally recommend physical therapy
	For patients with clinical manifestations of DADA2, we strongly recommend treatment with tumor necrosis inhibitors over GCs alone

*PAN* Polyarteritis nodosa, *GC* Glucocorticoids, *AZA* Azathioprine, *MTX* Methotrexate

Identification of coronary lesions in PAN is challenging. Computed tomography angiography (CTA) and coronary angiography (CAG) risk lesion identification without ability to define etiology (i.e., arteritis/inflammation vs. atherosclerosis). CAG with optical coherence (OC) is one method of differentiating arteritis opposed to atheroma but mandates invasive study [11]. CAG with intravascular ultrasound (IVUS) and cardiac magnetic resonance (CMR) are invasive and non-invasive methods of visualizing the vessel lumen. IVUS offers practical benefits during placement of interventional devices in a mixed stenotic-aneurysmal patterned vessel. Fluorodeoxyglucose-positron emission tomography (FDG-PET) reportedly approaches 92% and 100% sensitivity and specificity for large vessel vasculitis [28]. Less evidence supports FDG-PET in medium PSV’s making it unsurprising that FDG-PET scanning was negative in our patient. Nonetheless, detecting metabolic uptake as a marker of inflammatory change that precedes anatomic or functional disturbance captured in CAG, CT and MRI could potentiate medical interventions.

Subsidiary the uncommon nature of coronary PAN, comment on treatment and therapy remains guarded [29]. Strategies include immunosuppressive therapy (ISx) alone and ISx with re-vascularization. These recommendations, not specific to coronary involvement, are provided by the ACR and include glucocorticoids (GC) with Cyclophosphamide (Cyc) as first line, with substitution of (Cyc) for non-GC ISx agents (I.e., Azathioprine, Methotrexate) in non-severe disease [7] (Table 3). Therapy duration was studied by (Guillevin and colleagues) who noted 12 doses of monthly Cyc along with GC is superior to a 6-month strategy, indicated by higher survival (HR 0.44, p 0.02) and sustained remission (HR 0.34, p 0.02) at 32-month follow-up [30].

Important to coronary PAN, therapeutic consideration includes thrombosis risk. Aside from the risk associated with generalized vascular inflammation, other works show patients with coronary PAN circulate anti-cardiolipin antibodies in greater frequency (~ twofold) than patients without coronary lesions [4]. Further, the historic necropsy studies revealed cases of thrombus within aneurysmal arterial segments [9, 10]. Mechanistically, acute myocardial infarction (AMI) in PAN may occur by atherosclerotic deposition resulting in acute plaque rupture similarly to AMI in the general population, but complete thrombotic occlusion in the absence of atherosclerotic pathology has also occurred [31]. Preventative therapy should be provided for all patients not having excessive risk of rupture.

Revascularization may be indicated when patients' symptoms or acuity are incompatible with timelines of medical therapy. (Table 4) highlights the literature's prior revascularization cases and outcomes; clearly, there remains paucity in both quantity and longitudinal follow-up regarding outcomes. Importantly, the safety of CAG in actively inflamed vessels is proven, dating back to 1981 and should not steer physicians away from meaningful interventional opportunities [17].

**Table 4:** Review of interventions and outcomes in prior literature

Study	Presentation Inflammatory Markers	Coronary Angiography	Intervention	Other Systemic Arterial Disease	Post-Intervention Inflammatory Markers	Followup	Adjunctive Therapy
Lai [4]	NR	NR	1. stent	NR	ESR normal	Stent restenosis at 1 year	GC & Cyc
[4]	NR	NR	1. stent	NR	NR	Patent stent at 1 year	GC & Cyc
[4]	NR	1. Left Main trunk aneurysm 20 × 14 mm	1. CABG^a^	NR	CRP normal ESR normal	Death post-operative day 2 after IVC rupture	GC & Cyc
Huang [12]	CRP normal ESR normal	1. Plaque infiltration of LMC 2. Occlusion of all the three major coronary arteries, with multiple aneurysms 3. 95% stenosis of the OM	1. stent	1. Multiple stenoses, occlusion and aneurysms of renal arteries	CRP normal ESR normal	9-month echocardiogram without evidence of decline in function or structure	1. Pred & Cyc initially transitioned to AZA for maintenance prior to intervention 2. Pred & MTX post-intervention
Yanagawa [2]	CRP normal ESR normal	1.Aneurysmal, left coronary vessels with multiple stenoses 2. 90% lesions of pLAD, stented D, pLCx and OM2 3.pRCA had 70% lesion	1. BMS to mRCA 2. DES to in-stent stenosis of RCA + BM stent to D1 3. CABG: LIMA-LAD and SVG-D1-OM-PDA	1. bilateral renal artery aneurysms 2. perinephric hemorrhage	NR	NR	1.Pred & Cyc prior to CABG
Ucar [32]							
Yamamoto [22]	CRP normal ESR normal	1. Aneurysm of LMC, LAD, and LCx 2. pRCA total occlusion 3. 99% lesions in LAD and PL branch	1.CABG: SVG-LAD & SVG-PL^b^	1.Superior mesenteric artery aneurysm 2. Bilateral renal arteries aneurysm 3.Tortuose abdominal aortic artery 3.occlusion vs. Sev. Stenosis of r. gastroepiploic A 4. Stenotic proximal LITA & occluded RITA in middle segment 5. Bilateral radial A. aneurysms	NR	Post-operative CTA showed occlusion of SVG-PL	1. Prednisolone at Dx, d/c’d 8 years before presentation
Erbersberger [33]		1. 30 mm aneurysm of the RCA	1. CABG: SVG-RCA	1. inguinal aneurysm			1. post-operative Cyc
Reindl [31]	CRP 196 mg/l	1. Complete thrombotic occlusion of RCA 2. Absence of atherosclerosis in CA’s	1. stent	1. Bilateral renal infarctions and aneurysms 2. splenic infarctions 3.)cerebral infarctions 4.) popliteal occlusions	NR	NR	1. methylprednisolone administered prior to intervention; continued unnamed immunosuppression post-intervention
Canoplat [20]	NR	1. Coronary ectasia in LAD & LCx 2. 100% RCA occlusion due to dissection & 2 distal consecutive thrombotic lesions	1. Bare metal stent to distal RCA followed by bare metal stent to mid RCA	1. Left Renal and axillary aneurysms	CRP and ESR normal	6- and 12-month myocardial scintigraphy negative for ischemia	Cyc daily and Prednisolone every other day prior to presentation; no changes thereafter
Lewandowski [11]	NR	1.) LAD and RCA stenosis without typical atherosclerotic features 2. NS – performed at OSH 3.) stable LAD stenosis, new mRCA stenosis, new neointimal layer covering initial stents	1.) DES stent to LAD and RCA 2.) LCx bioresorbable stent 3.) repeat DES stenting of the LAD and RCA	NR	NR	NR	1. LD prednisone daily prior to presentation 2. methylprednisolone and Cyc after LCx stent 3. continuation of #2
Wagner [34]	ESR 120 mm/1st h CRP 306.3 mg/l	1. 90% LAD lesion with multiple distal occlusions	1. Stent	1.Whitematter hypo-intensities on MRI	NR	NR	1. Methylprednisolone & Cyc transitioned to prednisolone & Cyc prior to intervention 2. Prednisolone &Cyc post-intervention
Bayturan [29]	NR	1. CTO of prior RCA stent with bridging collaterals 2. Non-obstructive LCx lesions 3. 40-45 mm aneurysm of the LAD OM1	1. Endovascular coil embolization	1.Popliteal A. aneurysm	NR	Asymptomatic at 6 weeks; CAG at 6 weeks showed stable aneurysm	NR
Yuji [25]	CRP normal	1. RCA obstructed by thrombus within aneurysm 2. LAD & LCx markedly stenotic, thrombus within aneurysms 3. Collateral development between AV node branch & PD branch	1. CABG: bilateral IMA’s	1. Intestinal ischemia (thrombosis to supra mesenteric A.) 2. Positive Allen’s testing of radial arteries	NR	NR	1. GC & AZA prior to procedure
**Present Study**	hsCRP 5 mg/l ESR 49 mm/h	1. 90% stenosis of the LAD 2. 70% of the 2nd diagonal branch 3. 100% of the 1st obtuse marginal 4. 70% of the LCx 5. 90% RCA occlusion	1. CABG: LIMA-LAD and SVG-OM-LPDA-RPDA	1. Splenic infarction 2. Abdominal Aortic Aneurysm (infrarenal) 3. Bilateral Femoral Artery Aneurysm 4. Celiac Axis & Hepatic Artery Stenosis	NR	Asymptomatic at 1 month	1. Methylprednisolone transitioned to Pred prior to procedure 2. Pred & Cyc post-intervention

*Abbreviations*: *AZA* Azathioprine, *BMS* Bare Metal Stent, *CABG* Coronary Artery Bypass Graft, *CTA* Computed Tomography Angiography, *Cyc* Cyclophosphamide, *D* Diagonal, *DES* Drug-Eluting Stent, *GC* Glucocorticoids, *NR* Not Reported, *LAD* Left Anterior Descending, *LCx* Left Circumflex, *LD* Low Dose, *LIMA* Left Internal Mammary Artery, *LMC* Left Main Coronary, *LPDA* Left Posterior Descending Artery, *m* Mid, *MTX* Methotrexate, *OM* Obtuse Marginal, *PD* Posterior Descending, *PL* Posterolateral, *Pred* Prednisone, *p* Proximal, *RCA* Right Coronary Artery, *RPDA* Right Posterior Descending Artery, *SVG* Saphenous Vein Graft

^a^graft not stated

^b^LIMA aborted intraoperatively after harvest

Of the 19 patients studied by (Lai et al.), 3 received intervention (2 DES and 1 CABG). At 1 year follow-up, 1 patient with DES had in-stent restenosis while the other retained stent patency [4] (Table 4). (Huang et al.) contributed the case of a 22-year-old male with 3-vessel stenosis presenting with AMI, receiving stent placement to the OM1 [12]. Four additional stent placements were detailed in the literature. PCI intervention has ranged from 1 to 3-vessel disease requiring multi-stage angioplasty (Table 4). Generally, PCI achieves revascularization and resolution of symptoms, albeit limited longitudinal follow-up. Confounding the good outcomes are single case incidents of in-stent restenosis at 1 year and rapid neo-endothelization within 2 previously placed DES’s [4, 11]. In review, both authors raise the issue of placement technique and stent apposition difficulties as possible culprits to such outcomes. Adequacy of concomitant immunosuppressive regimen is another consideration. The role of which may be illuminated by the absent neo-endothelization after placement of a second set of stents with more intensive adjunct medical therapy [11].

Treatment of non-occlusive lesions (aneurysm, dissection) is equally imperative owing to risk of rupture, fistula, thromboembolism and angina [29, 35]. Medical therapy reduced aneurysm size in non-coronary lesions. Additionally, follow-up study of 4 prospective trials showed GC & Isx therapy prolong survival in patients with Five Factor Scores (FFS) > 2 [36]. PCI with stent placement is the mainstay of aneurysm therapy but stent apposition in vessels with multiple points of aneurysm and stenosis is technically challenging and not well studied, disturbing current interventional knowledge. Endovascular coiling is another modality, and there is reported success in alleviating anginal symptoms in a patient with a large LAD CAA [29]. Techniques such as these may offer additional benefits as cases accumulate.

The surgical approach to therapy has generated mixed results and raises concern over graft vessel candidate(s) [2, 6, 22]. For instance, in Kawasaki disease, left internal mammary artery (LIMA) graft during coronary artery bypass graft (CABG) procedures performed in adults demonstrate 15-year patency at rates as high as 91% [37]. As such, consensus among surgeons has been preference for LIMA over other vessels. Sparse literature and absent longitudinal study prevent such insight for PAN. Takayasu arteritis (TA) offers another example. Here, IMA’s are avoided in favor of saphenous vein grafts (SVG) because subclavian stenosis, has been associated with TA and would compromise flow through a LIMA. In PAN, one case has documented distal aneurysm and occlusion in the axillary and brachial arteries of a patient with coronary PAN, but no report of subclavian disease exists [38]. Others described IMA disease in females, introducing concern for graft failure in IMA anastomosis [39]. Further confounding graft selection is report of an IMA graft artery without significant stenosis on CTA discovered to be markedly stenotic intraoperatively, culminating in abortion of vessel harvest [6, 22].

Both the pathology and anatomic location of PAN tempt comment regarding preference for utilization of venous grafts which might avoid direct insult by arteritis. Optimism for this strategy is offset by bypass surgery where 1 patient died after IVC rupture on post-operative day #2 despite laboratory values lacking inflammation [4]. This event reinforces the diffuse and systemic nature of such inflammatory states. In the past, successful grafts used the LIMA and SVG [2, 24] and SVG alone [22, 32, 33]. In each surgical revascularization, preoperative inflammation level was normal, assessed by CRP and ESR. Making the prudent point that those treated with revascularization should also receive medical therapy to reduce inflammatory burden and perhaps breeding the question of whether recent updates in CAD therapies to include colchicine may have synergistic utility in the PAN population. Failure of suppression may increase risk of graft failure or delay healing.

The first case to utilize bilateral IMA’s as graft material was recently reported [6]. A 21-year-old female diagnosed with PAN 1 after an event of intestinal ischemia was clinically asymptomatic from a cardiac perspective, but coronary CT revealed 3-vessel aneurysm, stenosis and intramural thrombus. Decision for CABG was complicated by positive Allen’s test and anatomic length preventing graft of the radial artery and gastroepiploic artery, respectively. Yet, the patients' age was felt to necessitate use of arterial material. LIMA-LAD with T-composite anastomosis to RIMA-OM1-PDA was successful.

### Summary

Polyarteritis Nodosa is a rare SVD predominantly affecting medium arteries. Previous teaching and intervention focused on renal, neurological, gastrointestinal and cutaneous involvements, while coronary involvement was considered rare. Accumulating evidence suggests coronary arteritis confers significant morbidity and mortality to these patients. CAG remains the standard diagnostic tool, while new modalities such as OC may be required to differentiate inflammatory change from atherosclerotic process. Identifying risk for coronary involvement (new hypertension, celiac involvement) assists physicians in screening appropriate populations. Standard atherosclerotic risk factors do not approximate risk for coronary disease. PCI with stenting is preferred therapy for occlusive and aneurysmal disease. CABG is another option, especially in 3-vessel disease, but limited case numbers portend careful graft selection. Optimal interventional strategy includes preoperative reduction of inflammatory burden followed by post-operative ISx in conjunction with anti-coagulation.

Herein, we report a case of 3-vessel occlusive disease culminating in a 4-vessel CABG schema not available in prior literature. Though recent works carried our knowledge forward, significant opportunities to advance clinical practice in population screening, interventional materials and optimization of medical management abound.

## Data Availability

All data generated or analysed during this study are included in this published article.

## Abbreviations

AMI: Acute myocardial infarction
AZA: Azathioprine
BMS: Bare metal stent
CABG: Coronary artery bypass graft
CAD: Coronary artery disease
CTA: Computed tomography angiography
Cyc: Cyclophosphamide
D: Diagonal
DES: Drug-eluting stent
FDG-PET: Fluorodeoxyglucose-positron emission tomography
FFS: Five factor score
GC: Glucocorticoids
IMA: Internal mammary artery
ISx: Immunosuppression
IV-US: Intravascular ultrasound
NR: Not Reported
LAD: Left anterior descending
LCx: Left circumflex
LD: Low dose
LHC: Left heart catheterization
LIMA: Left internal mammary artery
LMC: Left main coronary
LPDA: Left posterior descending artery
m: Mid
MINOCA: Myocardial Infarction with non-obstructed coronary arteries
MTX: Methotrexate
OC: Optical coherence
OM: Obtuse marginal
p: Proximal
PCI: Percutaneous coronary intervention
PD: Posterior descending
PL: Posterolateral
Pred: Prednisone
PSV: Primary systemic vascular disease
RCA: Right coronary artery
RPDA: Right posterior descending artery
SV: Systemic vasculitide
SVD: Systemic vascular disease
SVG: Saphenous vein graft
TA: Takayasu's arteritis
TTE: Transthoracic echocardiogram
WMA: Wall motion abnormalities
